# Methane emission from a cool brown dwarf

**DOI:** 10.1038/s41586-024-07190-w

**Published:** 2024-04-17

**Authors:** Jacqueline K. Faherty, Ben Burningham, Jonathan Gagné, Genaro Suárez, Johanna M. Vos, Sherelyn Alejandro Merchan, Caroline V. Morley, Melanie Rowland, Brianna Lacy, Rocio Kiman, Dan Caselden, J. Davy Kirkpatrick, Aaron Meisner, Adam C. Schneider, Marc Jason Kuchner, Daniella Carolina Bardalez Gagliuffi, Charles Beichman, Peter Eisenhardt, Christopher R. Gelino, Ehsan Gharib-Nezhad, Eileen Gonzales, Federico Marocco, Austin James Rothermich, Niall Whiteford

**Affiliations:** 1 Department of Astrophysics, American Museum of Natural History, New York, NY USA; 2 Department of Physics, The Graduate Center City University of New York, New York, NY USA; 3 Department of Physics, Astronomy and Mathematics, University of Hertfordshire, Hatfield, UK; 4 Planétarium Rio Tinto Alcan, Montreal, Quebec Canada; 5 Département de Physique, Université de Montréal, Montreal, Quebec Canada; 6 School of Physics, Trinity College Dublin, The University of Dublin, Dublin, Ireland; 7 Department of Physics & Astronomy, Hunter College, New York, NY USA; 8 Department of Astronomy, University of Texas at Austin, Austin, TX USA; 9 Department of Astronomy, California Institute of Technology, Pasadena, CA USA; 10 IPAC, Caltech, Pasadena, CA USA; 11 NSF’s National Optical-Infrared Astronomy Research Laboratory, Tucson, AZ USA; 12 United States Naval Observatory, Flagstaff, AZ USA; 13 Exoplanets and Stellar Astrophysics Laboratory, NASA Goddard Space Flight Center, Greenbelt, MD USA; 14 Department of Physics & Astronomy, Amherst College, Amherst, MA USA; 15 Jet Propulsion Laboratory, California Institute of Technology, Pasadena, CA USA; 16 NASA Ames Research Center, Mountain View, CA USA; 17 Department of Physics, San Francisco State University, San Francisco, CA USA; 18 Department of Astronomy and Carl Sagan Institute, Cornell University, Ithaca, NY USA

**Keywords:** Stars, Exoplanets

## Abstract

Beyond our Solar System, aurorae have been inferred from radio observations of isolated brown dwarfs^1,2^. Within our Solar System, giant planets have auroral emission with signatures across the electromagnetic spectrum including infrared emission of H_3_^+^ and methane. Isolated brown dwarfs with auroral signatures in the radio have been searched for corresponding infrared features, but only null detections have been reported^3^. CWISEP J193518.59-154620.3. (W1935 for short) is an isolated brown dwarf with a temperature of approximately 482 K. Here we report James Webb Space Telescope observations of strong methane emission from W1935 at 3.326 μm. Atmospheric modelling leads us to conclude that a temperature inversion of approximately 300 K centred at 1–10 mbar replicates the feature. This represents an atmospheric temperature inversion for a Jupiter-like atmosphere without irradiation from a host star. A plausible explanation for the strong inversion is heating by auroral processes, although other internal and external dynamical processes cannot be ruled out. The best-fitting model rules out the contribution of H_3_^+^ emission, which is prominent in Solar System gas giants. However, this is consistent with rapid destruction of H_3_^+^ at the higher pressure where the W1935 emission originates^4^.

## Abstract (web summary)

Methane emission from a very cool brown dwarf, perhaps arising from an aurora, has been detected in James Webb Space Telescope observations.

## Main

Brown dwarfs are a class of object that links planetary and stellar astrophysics. They have temperatures between approximately 3,000 and 250 K and spectral classifications of L, T and Y (refs. ^5,6^). The Y dwarfs are a recent addition to our assortment of known compact objects. They are probably the coldest sources that formed through the star formation process^7^. These cold objects are directly comparable to Jupiter, with the coldest known Y dwarf—WISE J085510.83-071442.5—having a temperature of approximately 250 K (ref. ^8^), just 100 K warmer than Jupiter^9^. Y dwarfs present an extraordinary observational challenge for ground-based telescopes given their intrinsic faintness and need for infrared instrumentation^10–12^. The James Webb Space Telescope (JWST), a space-based 6.5 m infrared observatory, is perfectly suited for revolutionizing our understanding of brown dwarfs and in turn exo-Jupiter atmospheres^13^. In this work we report observations of two brown dwarfs obtained with JWST cycle 1 Guest Observer (GO) programme 2124. We have obtained NIRSpec G395H spectra and mid-infrared F1000W, F1280W and F1800W photometry from the Mid-Infrared Instrument (MIRI) on JWST for the Y dwarfs CWISEP J193518.59-154620.3 (W1935 for short) and WISE J222055.31-362817.4 (W2220 for short).

We combined all literature data on these two objects alongside the JWST data for each source and created absolute spectral energy distributions (SEDs), which we could compare and contrast. By integrating over the SEDs using the open-source code SEDkit (ref. ^14^), we find that the luminosity ratio to the Sun (*L*_bol_/*L*_⊙_) for W2220 and W1935 are identical within uncertainties, with values of log(*L*_bol_/*L*_⊙_) equal to −6.4 ± 0.1 and −6.3 ± 0.1, respectively. Neither source has any age indications, so we assumed a conservative age range of 4.5 ± 4.0 Gyr, which we used to semi-empirically calculate the following values for W1935 and W2220, respectively: radius in Jupiter radii (*R*_Jup_) of 0.95 ± 0.14 *R*_Jup_ and 0.94 ± 0.14 *R*_Jup_; effective temperature (*T*_eff_) of 482 ± 38 K and 480 ± 41 K; surface gravity (log *g*) of 4.7 ± 0.5 dex (both); and mass in Jupiter masses (*M*_Jup_) of 6–35 *M*_Jup_ (both). Given that these objects are indistinguishable in their fundamental parameters, they are excellent objects for intercomparisons.

**Fig. 1:**
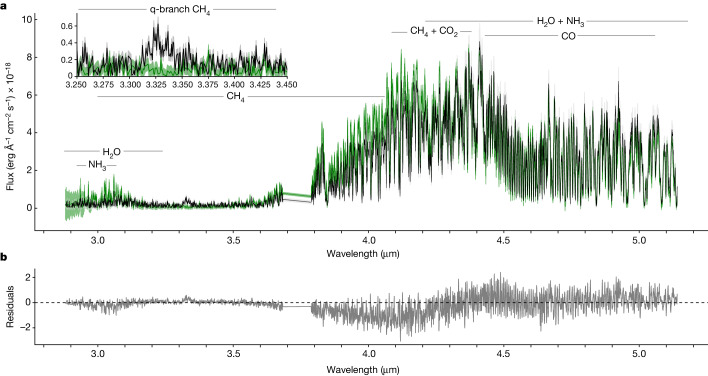
JWST NIRSpec G395H spectra for the Y dwarfs W1935 and W2220. **a**, NIRSpec G395H portion of the SED for W1935 (black) compared to that of W2220 (green). Shading for both sources represents the 1*σ* uncertainty on the flux. Major opacity sources are labelled. The inset is a zoom-in of the 3.326 μm CH_4_*ν*_3_ band. **b**, The residuals between the two spectra.

Spectroscopically, we found that W1935 and W2220 are near clones of each other, with both showing clear and strong signatures of CH_4_, CO, CO_2_, H_2_O and NH_3_ (Fig. 1). There is one visually striking difference between the spectral characteristics of the two sources. Although W2220 shows the expected CH_4_ q-branch absorption centred at 3.326 μm, W1935 shows emission over that same wavelength range (see inset for the 3.25–3.45 μm area in Fig. 1).

To model the 3.326 μm emission feature in W1935 as well as compare and contrast with W2220, we used the Brewster retrieval framework^15,16^, which has successfully retrieved the properties of numerous brown dwarfs^17–19^. For our baseline model for both objects, we assumed a cloud-free atmosphere. Alongside continuum opacity sources described in detail in refs. ^15,16^, we included the following gases as absorbers, as they would be expected to have an impact in the wavelength range covered by our data: H_2_O, CH_4_, CO, CO_2_, NH_3_, H_2_S and PH_3_. The power of a retrieval is in its ability to parameterize the temperature–pressure (*T*/*P*) profile, thus giving an insight into the energy distribution within a given atmosphere. Brewster can do this in a flexible way, without prescribing that the atmosphere be in radiative–convective equilibrium or have a particular slope. This gives the *T*/*P* profile freedom to adopt whatever shape is justified by the data, including an inversion where the temperature increases with altitude.

**Fig. 2:**
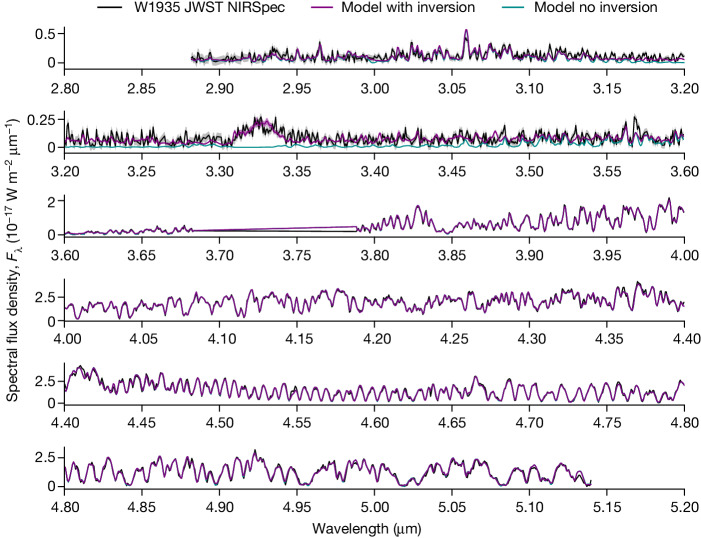
JWST G395H spectrum for W1935 overlaid with the best-fitting models with and without temperature inversion. Overlaid in purple and dark cyan are the median retrieval models for the source with and without temperature inversion, respectively. Data uncertainties are shaded in grey. The 67% confidence intervals in the model posteriors are shaded in dark cyan and purple but are of comparable extent to the width of the plotted data.

**Fig. 3:**
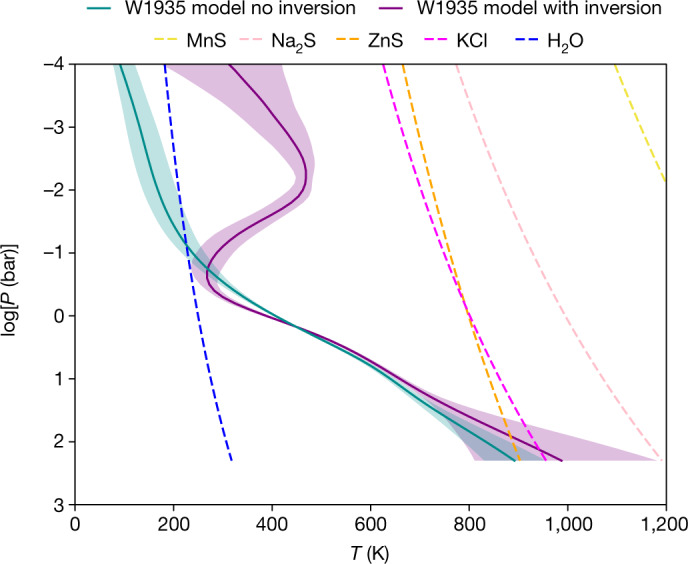
Retrieved thermal profiles for W1935 with and without thermal inversion. Median posterior profiles for the models are shown in purple and dark cyan, respectively. The 67% confidence intervals for the model posteriors are indicated with shading for each. Also plotted with dashed lines are the condensation curves (assuming solar composition gas) for possible cloud species.

The results of the retrieval verified that the two sources were near clones in all abundances (Extended Data Table 2). However, the *T*/*P* profiles for the two sources show striking differences. Although the best-fitting retrieved *T*/*P* profile for W2220 is consistent with the temperature decreasing with increasing altitude throughout the atmosphere (Extended Data Fig. 3), as expected for an isolated source, the best-fitting retrieval for W1935 displays a temperature inversion of approximately 300 K centred at approximately 1–10 mbar (Fig. 3). As a result, alongside our baseline model, we tested a model that forbade an inversion. For W2220, the ‘no inversion’ result was indistinguishable from the base result. However, for W1935, the ‘no inversion’ retrieval could not reproduce the CH_4_ emission feature (Fig. 2). Hence, we conclude that the observed CH_4_ emission arises as a result of thermal inversion in the stratosphere of this cool, isolated brown dwarf.

Temperature inversions have been inferred before in substellar atmospheres, for both brown dwarfs^20^ and giant exoplanets^21^, not to mention being nearly ubiquitous within the Solar System. The common feature of all of these cases is the presence of an irradiating star. However, the stratospheres of the gas planets in the Solar System display temperatures even higher than can be attributed to irradiation alone^22–24^. Our result represents a spectacular extension of this gas giant phenomenon without any stellar irradiation.

Much work has been dedicated to understanding the Solar System cases of enhanced stratospheric heating. Both external heating by auroral processes and internal energy transport by vertically propagating waves from deeper in the atmosphere are possible mechanisms^25–27^. The latter is a plausible explanation for the thermal inversion modelled for W1935. However, one would expect this process to be ubiquitous across a range of atmospheres. Given that this is the only non-irradiated example to date, such a universal mechanism is less likely to be responsible.

External heating by auroral processes may be a more likely mechanism. Recent observations by ref. ^28^ indicate that the bulk of the heating in Jupiter’s upper atmosphere is driven by redistribution from hot auroral polar regions. In addition, alongside methane fluorescence from solar pumping, some Jovian methane emission has been tentatively attributed to heating by auroral processes^29^.

Ultracool dwarfs, which comprise the lowest-mass stars and substellar-mass objects, have long been surmised to host aurora akin to those found in the giant planets of our Solar System, like Jupiter and Saturn. Studies have shown that approximately 5% of ultracool dwarfs demonstrate highly circularly polarized, rotationally modulated, radio pulses attributed to the electron cyclotron maser instability, which is the mechanism responsible for auroral radius emission^1,30^. Such a low-detection rate suggests that any stratospheric heating arising from those auroral processes should be similarly rare.

As a further test for W1935, we implemented a retrieval that included H_3_^+^, a common emitter produced by aurorae in giant planets. Interestingly, the addition did not improve the fit for W1935 and yielded a null detection for H_3_^+^ emission. Although the thermal inversion in W1935 has a similar overlying column mass to the equivalent region in the Jovian atmosphere, the higher surface gravity results in a gas density that is approximately 100 times higher. At these densities, the lifetimes of H_3_^+^ ions are much shorter than the typical emission timescale^4^, so their absence is not surprising.

The detection of CH_4_ in the emission from an object with a mass range of 6–35 *M*_Jup_ and a temperature of 482 ± 38 K (approximately 300 K warmer than Jupiter) is enticing. Moreover, the appearance of a temperature inversion in an object that lacks an irradiating star compounds the interest. For Solar System giants with equivalent spectral emission and upper atmospheric heating, a contributing factor outside of solar pumping is auroral processes linked to nearby moons (Io for Jupiter and Enceladus for Saturn). Regardless of what is causing the thermal inversion on and consequent methane emission from W1935, this source represents an outstanding laboratory for investigating linked phenomena that are prominent in our own Solar System.

## Methods

### Sample

**Extended Data Fig. 1:**
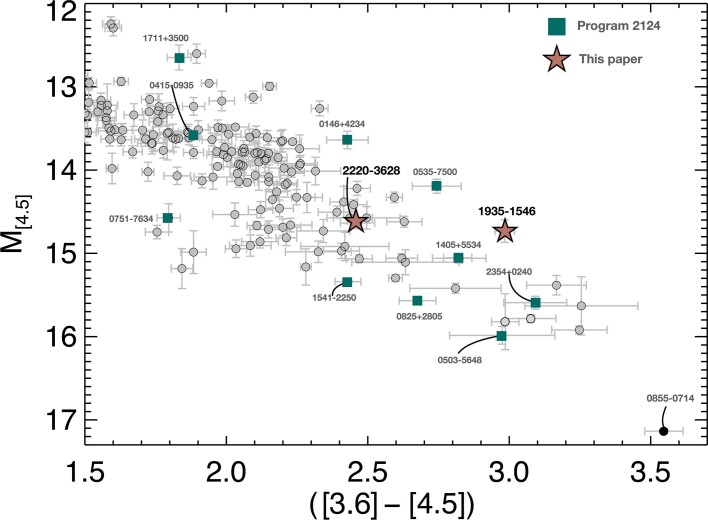
The Spitzer color-magnitude diagram for cold brown dwarfs. The sources in program 2124 are green filled squares. Both W2220 and W1935 are highlighted by a five-point star to emphasize their position. The 1*σ* unceratinty on both color and absolute magnitude is plotted. We also label and highlight as a black filled circle the coldest known brown dwarf 0855-0714 to demonstrate the extreme of Y dwarf parameters.

JWST cycle 1 GO programme 2124 (PI Faherty) is obtaining G395H NIRSpec spectra for 12 cold brown dwarfs that span late-type T and Y dwarf classes. Sources were selected by their position in the mid-infrared (Spitzer) colour–magnitude diagram (*M*_[4.5]_ versus [3.6]–[4.5] where [3.6] and [4.5] are two Spitzer bands). Four colour bins were roughly defined to represent changing temperatures (from 800 to less than 350 K). Three sources were chosen in each colour bin such that one source was close to the median property line for the population^7^. The other two were, respectively, the brightest and faintest in *M*_[4.5]_ for that colour bin. Two of the sources chosen were CWISEP J193518.59-154620.3 (W1935; ref. ^31^) and WISE J222055.31-362817.4 (W2220; ref. ^32^), which are the subject of this paper. At the time of the project design, we speculated that the [3.6]–[4.5] colour would define the temperature bin and we did not expect W1935 and W2220 to be so comparable. The results of this paper are strong evidence that *M*_[4.5]_ is a better temperature binning parameter. Extended Data Fig. 1 shows the Spitzer colour–magnitude diagram for cold brown dwarfs and highlights the sample for programme 2124 with the positions of W1935 and W2220 emphasized.

#### CWISEP J193518.59-154620.3

CWISEP J193518.59-154620.3 (W1935 for short) was first reported in ref. ^31^ after a concerted effort was applied to find cold compact sources within newly analysed Wide-field Infrared Survey Explorer (WISE) catalogue data^33,34^. The object’s discovery was the result of a collaboration between the CatWISE team and the citizen science project Backyard Worlds: Planet 9 (ref. ^35^). A Spitzer follow-up resulted in a Spitzer [3.6]–[4.5] colour of 3.24 ± 0.31 mag, making it one of the reddest mid-infrared sources known to date. Additional follow-up observations were done to obtain Spitzer data with a higher signal-to-noise ratio (S/N) in both channels, and the colour was refined to a Spitzer [3.6]–[4.5] colour of 2.984 ± 0.034 mag in ref. ^36^. Reference ^7^ reported that the parallax for this source was 69.3 ± 3.8 mas and that the total proper motion was 293.4 ± 16.3 mas yr^−1^. As noted by ref. ^36^, this source has a particularly low tangential velocity (vtan) compared to other Y dwarfs analysed. The estimated spectral type from its photometry and parallax was > Y1, and the temperature was predicted to be 367 ± 79 K (ref. ^7^). Reference ^37^ obtained Gemini NIRI imaging for the source and reported Mauna Kea Observatories (MKO) *J* = 23.93 ± 0.33 mag.

#### WISE J222055.31-362817.4

WISE J222055.31-362817.4 (W2220 for short) was first reported in ref. ^32^ after a search of the WISE catalogue^38^ for cold compact objects. Reference ^32^ followed this up with observations by the Spitzer Space Telescope, Keck/NIRSpec-N3 and Keck/NIRSpec-N5 as well as AAT/IRIS2 and SOAR/OSIRIS. The source was confirmed as a cold brown dwarf with WISE W1, W2, Spitzer [3.6], [4.5], and MKO *JH* photometry as well as near-infrared spectra. References ^39,40^ obtained a Hubble Space Telescope (HST) grism spectrum for the source, which confirmed the Y0 classification with higher S/N data. Follow-up photometric and astrometric imaging was done using both space-based (for example, Spitzer) and ground-based (for example, the FourStar Infrared Camera installed on Magellan) instruments and the most recent and highest S/N trigonometric parallax is 95.5 ± 2.1 mas (see refs. ^7,40–43^ for the astrometric history of the source). Reference ^7^ estimated a temperature of 452 ± 88 K for this source based on its position on the colour–magnitude diagram.

### The data

JWST programme 2124 obtained both NIRSpec G395H spectra and MIRI F1000W, F1280W, and F1800W photometry to fill out the peak of the SED and the Rayleigh–Jeans tail of the SED for 12 brown dwarfs. NIRSpec data were obtained using the F290LP filter, the G395H grating, the S200A1 aperture and the SUB2048 subarray. The resultant wavelength coverage ranged from 2.87 to 5.14 μm with a resolving power of approximately 2,700. Acquisition images were first obtained for each target using the WATA method, the CLEAR filter and the NRSRAPID readout pattern. W2220 was observed with NIRSpec on 4 November 2022 with 28 groups per integration, ten integrations per exposure and three dithers for a summation of 30 total integrations in 1,356 s of exposure time. The recorded time including the overhead for the W2220 NIRSpec observation was 1.42 h. W1935 was observed with NIRSpec on 17 October 2022 with 46 groups per integration, 11 integrations per exposure and three dithers for a summation of 33 total integrations in 2,417 s of exposure time. The recorded time including the overhead for the W1935 NIRSpec observation was 1.76 h.

MIRI photometry was obtained with the F1000W, F1280W, and F1800W filters. For each filter, the FASTR1 readout pattern was chosen with a two-point dither pattern. W1935 was observed with MIRI on 20 September 2022 using 15 groups per integration for F1000W, 13 groups per integration for F1280W and 11 groups per integration for F1800W. The total exposure time plus the overhead for the MIRI observing of W1935 was 1.03 h. W2220 was observed with MIRI on 18 October 2022 using seven groups per integration for F1000W, seven groups per integration for F1280W, and ten groups per integration for F1800W. The total exposure time plus the overhead for the MIRI observation of W2220 was 0.54 h.

As noted in ‘Sample’ above, both W2220 and W1935 have previously published photometry and W2220 has previously published spectra. Extended Data Table 1 lists all observables, both previous and new in this paper, included in the analysis that follows.

### Data reduction

We used the official JWST science calibration pipeline (v.1.10.0) to reduce the NIRSpec G395H spectra starting from uncalibrated data downloaded from the Mikulski Archive for Space Telescopes (MAST). The pipeline comprises three separate stages. Stage 1 performs detector-level corrections (for example, bias subtraction, dark subtraction and cosmic-ray detection) and ramp fitting to generate a count rate image for the individual uncalibrated image of each exposure. The resulting count rate images were calibrated by applying instrument-level and observing-mode corrections in Stage 2. Stage 3 combines several calibrated exposures and extracts the spectrum. We optimized the aperture extraction location by using the relative slit position of the target to account for inaccuracies in the object coordinates and the celestial World Coordinate System.

The flux uncertainties automatically propagated through the pipeline were all null for the extracted spectrum due to the most recent reference flat files for NIRSpec having NaN values. We recalculated the flux errors for the extracted spectrum by combining in quadrature the Poisson variance (FLUX_VAR_POISSON) and read noise variance (FLUX_VAR_RNOISE) provided in the extracted spectrum file.

Looking at the W1935 3.326 μm spectral feature alone, we carefully examined each dither position to confirm that the feature was present. Although this is, in general, an area of the spectrum with a low S/N (average of 5–10 across the entire feature), we found the methane emission persisted in individual single exposure dithers, thus confirming its presence.

For both W2220 and W1935, we used the MAST-produced pipeline reductions of MIRI photometry, choosing the aper total vegamag column as our preferred magnitude.

### Radial velocity

Given the high resolution of G395H data, we were able to compute radial velocities for both targets. All values reported are from a correlation with the models of ref. ^44^. Our values for W1935 and W2220, which are listed in Extended Data Table 1, are −36.9 ± 5.1 and −53.2 ± 2.8 km s^−1^, respectively. We used these values in the banyan sigma kinematic code^45^ to check whether our targets belonged to any known moving group. For both objects, the full kinematics yielded a 99.9% field population probability.

We also examined the total space velocity for each object and computed values of 42.02 ± 5.33 and 55.33 ± 2.82 km s^−1^ for W1935 and W2220, respectively. These values are consistent with the field brown dwarf population (see, for instance, ref. ^46^). Consequently, we have no evidence for youth or significant old age in the observables. Therefore, we choose a broad age range of 4.5 ± 4.0 Gyr to estimate fundamental parameters in the SED approach (see below).

### SED construction and results

Key to the analysis of this work was generating distance-calibrated SEDs inclusive of the newly obtained JWST data and all previously collected observables. To construct the SEDs, we used the open-source package SEDkit (ref. ^14^), which was first published and used by ref. ^47^ to analyse the fundamental parameters of brown dwarfs. As described in ref. ^47^, we first combined the parallax with spectra and photometry. For W1935, the only spectrum available was the G395H data. For W2220, an HST grism spectrum was available from ref. ^39^ as well as the newly acquired JWST data. All photometry used in the SED for both objects is listed in Extended Data Table 1.

Using SEDkit and the input data, we constructed the SEDs, integrated under the data as described in ref. ^47^ and calculated the *L*_bol_ value for each object. To examine the similarities and differences for the two objects, Fig. 1 shows the output SEDs for both W2220 and W1935 scaled to 10 pc. As described in the main text, the age estimate was paired with the evolutionary models of ref. ^48^ to acquire a radius range, and we then semi-empirically calculated *T*_eff_, log *g* and mass. All values are listed in Extended Data Table 1.

Figure 1 shows the G395H portion of the SEDs overplotted. The SEDs are both excellent fits for each other but show important differences. The 3.326 μm CH_4_ band is striking given that it is clearly in the emission from W1935 but absent for W2220. There is also a difference in the 3.8–4.3 μm region, where W2220 appears to have the same molecular features (see Figs. 2 and 3 and Extended Data Figs. 4 and 5), but the gas is warmer. Hence, the flux is higher for W2220. which drives its bluer mid-infrared colour.

### Retrieval analysis

We carried out retrieval analysis of the NIRSpec spectroscopy of our two targets using the Brewster package. Brewster is publicly available and was developed to model substellar atmospheres. It has been successfully applied to a range of brown dwarf atmospheres from cool T dwarfs to hot L-type subdwarfs, including cloudy and planetary mass objects^15–19,49,50^. This is its first application in the Y dwarf regime.

#### Retrieval method

Brewster consists of a forward model and sampler. The forward model produces a synthetic spectrum for a given set of atmospheric parameters. This is coupled to a Bayesian sampler, which explores the parameter space and estimates the posterior distribution for the forward model parameters given the data.

The forward model is a one-dimensional, radiative-transfer, layered-atmosphere, model consisting of 64 layers distributed uniformly in log pressure space (in bars) between 2.3 and −4.0. The model evaluates the emergent flux using the two-stream source function technique of ref. ^51^, including scattering^52^. This requires each layer in the atmosphere to be specified in terms of the wavelength-dependent optical depth, single scattering albedo and asymmetry parameter, as well as the temperature of the gas in the layer. The resultant spectrum is then processed to allow direct comparison to the data and the calculation of a likelihood for the fit.

The parameters for the forward model fall into the following groups: gas-phase opacity, cloud opacity, temperature structure and global properties of the target. The gas-phase opacity is set by the choice of absorbing gases, their concentration and distribution in the model atmosphere. We included the following gases in this study: H_2_O, CH_4_, CO, CO_2_, NH_3_, H_2_S and PH_3_. In this case, we parameterized the concentrations of the absorbing gases as vertically constant volume mixing ratios as in previous studies with this code. This approach provides substantial flexibility in arriving at possible solutions while still retaining computational simplicity. We neglected cloud opacity in this study and do not discuss this aspect of Brewster any further here but leave that work for a future study.

We modelled the temperature structure using the same method as refs. ^53,54^. Briefly, we parameterized the temperature at 13 evenly spaced points in log pressure and evaluated the temperature in our atmospheric layers using spline interpolation from these. We avoided implausible discontinuities and so-called ringing by penalizing the second derivative of the thermal profile in the likelihood by using the following log prior on the temperature:

1 $${\rm{ln}}p({\bf{T}})=-\frac{1}{2\gamma }\mathop{\sum }\limits_{i=1}^{N}{({T}_{i+1}-2{T}_{i}+{T}_{i-1})}^{2}-\frac{1}{2}{\rm{ln}}(2{\rm{\pi }}\gamma ).$$

This sums the discrete second derivative of the temperature *T* at each level *i*, and weights it by *γ*. The parameter *γ* sets the degree to which the likelihood will be penalized by wiggles in the thermal profile and is included in our retrieval as a free parameter. By including this free parameter, the data can set the degree of smoothing imposed on the profile. We follow ref. ^53^ in adopting an inverse *Γ* distribution as the hyperprior for *γ*, with properties specified in Extended Data Table 3.

We parameterized the global properties of the target as follows. The radius of the source is encoded in the scale factor that is applied to the top-of-atmosphere flux produced by our radiative-transfer code to allow comparison to the flux received by NIRSpec. This scale factor is equal to *R*^2^/*D*^2^, where *R* is the radius of the source and *D* is its heliocentric distance. Our forward model uses *R*^2^/*D*^2^, and hence, this is the quantity that is estimated directly by the retrieval. We used our knowledge of the target’s distance to estimate the radius post hoc and incorporated the uncertainties in both the distance and the absolute flux calibration of the spectrum in the error estimate for the radius. The surface gravity for the source is parameterized as log *g*, where *g* is the gravitational acceleration at the altitude of our model atmosphere in cm s^−2^. We combined this parameter with our post hoc estimate for the radius to estimate the mass of the source. Finally, we included parameters to apply rotational broadening and radial velocity shifts to our model spectrum. We used the rotational broadening code provided by ref. ^55^ to achieve the former.

In addition to the free parameters described above, we hardcoded the following into our model atmosphere. In addition to the absorbing gases, we assumed that the atmosphere is dominated by H_2_ gas, with He present at a solar H/He ratio. The total abundance of H_2_/He was set by the remaining fraction after the absorbing gas fractions have been accounted for.

We calculated layer optical depths due to the absorbing gases using opacities sampled at a resolving power *R* = 30,000 taken from the compendium of refs. ^56,57^, with updated opacities described in ref. ^15^ using the same method as ref. ^16^. We generated the latest version of CH_4_ line list^58^ with broadening coefficients relevant to H_2_/He atmospheres using the computational methodology of ref. ^59^ and incorporated PH_3_ opacities from refs. ^48,60^. We also included continuum opacities for H_2_–H_2_ and H_2_–He collisionally induced absorption using the cross sections from refs. ^61,62^ and Rayleigh scattering due to H_2_, He and CH_4_ but neglected the remaining gases. We also included continuum opacities due to bound–free and free–free absorption by H^−^ (refs. ^63,64^) and free–free absorption by H_2_^−^ (refs. ^65^).

The emergent spectrum was convolved with a Gaussian kernel of width 2.2 pixels to simulate the instrumental profile of the NIRSpec G395H.

In this work, we coupled the forward model to the emcee sampler^66^, which is the same method used in refs. ^15–18,49^. We used a log-likelihood function to assess the fit of the data to the model:

2 $${\rm{ln}}{\mathcal{L}}({\bf{y}}| {\bf{x}})=-\frac{1}{2}\mathop{\sum }\limits_{i=1}^{n}\frac{{({y}_{i}-{F}_{i}({\bf{x}}))}^{2}}{{s}_{i}^{2}}-\frac{1}{2}{\rm{ln}}(2{\rm{\pi }}{s}_{i}^{2}),$$

where the index *i* refers to the *i*th of *n* spectral flux points *y*_*i*_ with errors *s*_*i*_, which are compared to the forward model fluxes *F*_*i*_ for the current parameter set **x**. To (at least partially) account for model and other unaccounted sources of uncertainties, we inflated our errors using a tolerance parameter, such that our data error *s*_*i*_ is given by:

3 $${s}_{i}^{2}={\sigma }_{i}^{2}+1{0}^{b},$$

where *σ*_*i*_ is the measured error for the *i*th flux measurement and *b* is our tolerance parameter, which is retrieved^66–68^. The full set of parameters used and their priors are listed in Extended Data Table 3.

We used 16 walkers per dimension. Following 10,000 iterations of burn-in, we ran our chains for blocks of 30,000 iterations, checking each time for convergence. In all cases convergence appeared to be achieved after the first 30,000 block, but we ran an additional 30,000 in each case nonetheless.

#### Retrieval results

We ran three models each for W1935 and W2220: (1) our baseline model as described earlier, (2) the baseline model adjusted with an additional prior such that *T*_*i*_ < *T*_*i*+1_, which excludes the possibility of a temperature inversion and (3) the baseline model adjusted to include H_3_^+^ as an absorbing gas at pressures less than 1 mbar. To distinguish the preferred model given our data, we calculated the Bayesian information criterion (BIC) for each case. For a set of models, the one with the smallest (typically most negative) BIC will be the preferred model with the strength of the preference dependent on the value of the difference in the BICs, ΔBIC. By convention, the ‘winning’ model is defined as having ΔBIC = 0 and lower-ranked models have ΔBIC quoted relative to that. Reference ^69^ provided the following intervals for selecting between two models under the BIC:

- 0 < ΔBIC < 2: no preference worth mentioning;
- 2 < ΔBIC < 6: positive;
- 6 < ΔBIC < 10: strong;
- 10 < ΔBIC: very strong.

We found that for W1935, the baseline model is very strongly preferred over the model that includes H_3_^+^. Both of these models are very strongly preferred over the model that rules out a temperature inversion. By contrast, for W2220 we found that there is no preference worth mentioning between models with and without a temperature inversion, which are both strongly preferred over the model that includes H_3_^+^. This difference arises because W1935 shows emission in the CH_4_ q-branch at 3.326 μm whereas W2220 does not. The ΔBIC values for each model are given in Extended Data Table 4.

Note that the inclusion of H_3_^+^ in the model had no impact on the quality of the fit, the maximum likelihood or the values of the other parameters. The abundance of H_3_^+^ was an upper limit of $\log \,{f}_{{{\rm{H}}}_{3}^{+}}\approx -6$. We, thus, conclude that H_3_^+^ is not detected in either source.

**Extended Data Fig. 2:**
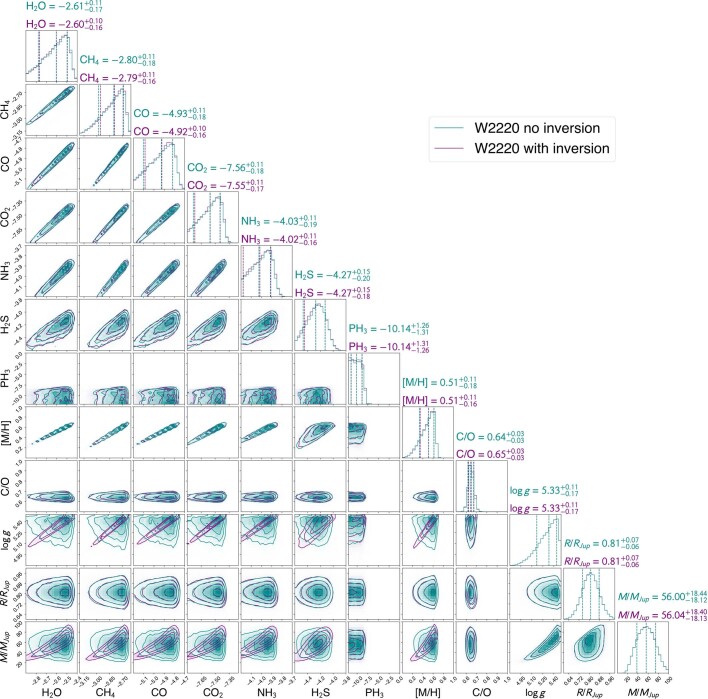
The retrieved composition of W2220, along with gravity, and derived quantities for mass, radius, metallicity and C/O. Posteriors for models with and without a temperature inversion are shown in purple and dark cyan respectively.

Parameter estimates (not related to the temperature structure) for W2220 are indistinguishable between the models that allow for a temperature inversion and those that do not. A comparison of the posterior distributions for models with and without a temperature inversion is shown in a corner plot in Extended Data Fig. 2.

**Extended Data Fig. 3:**
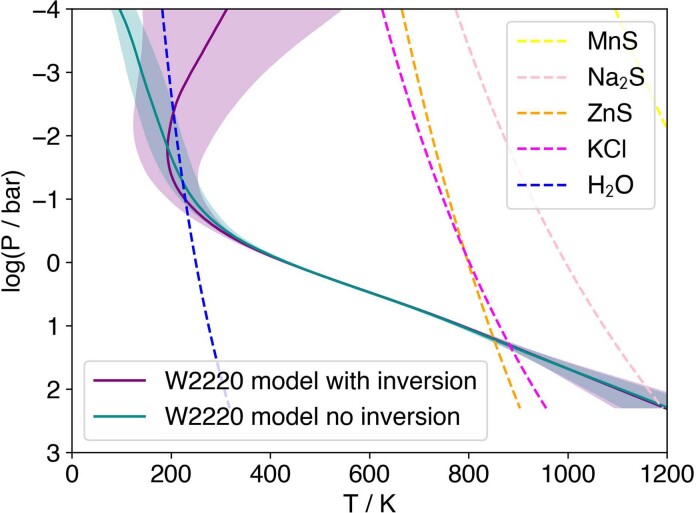
The retrieved thermal profiles for W2220 with and without an inversion. Median posterior T/P profiles for the best fit model without an inversion, and the lower ranked model that allowed an inversion are shown in dark cyan and purple respectively. The 67% confidence intervals in model posteriors are indicated with shading for each. Also plotted with dashed lines are the condensation curves (assuming Solar composition gas) for possible cloud species.

**Extended Data Fig. 4:**
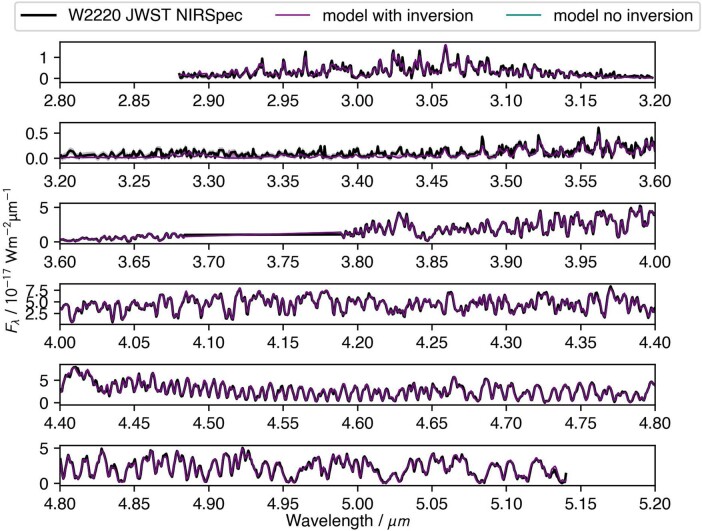
The JWST G395H spectrum for W2220. Overlaid in purple and dark cyan are the median retrieval models for the source with and without temperature inversions. Data uncertainties are shaded in grey. The 67% confidence intervals in the model posteriors are shaded in dark cyan and purple, but are of comparable extent to the line width.

The thermal profiles are also extremely similar, with only nonsignificant differences between the two. The two retrieved thermal profiles are compared in Extended Data Fig. 3. The two profiles are identical at pressures deeper than $\log [P\,({\rm{bar}})]=-2.0$ and do not deviate significantly from one another at shallower pressures. Extended Data Fig. 4 shows that the retrieved model spectra are also indistinguishable and a similarly good fit to the data as the baseline model for W1935 (Fig. 2).

**Extended Data Fig. 5:**
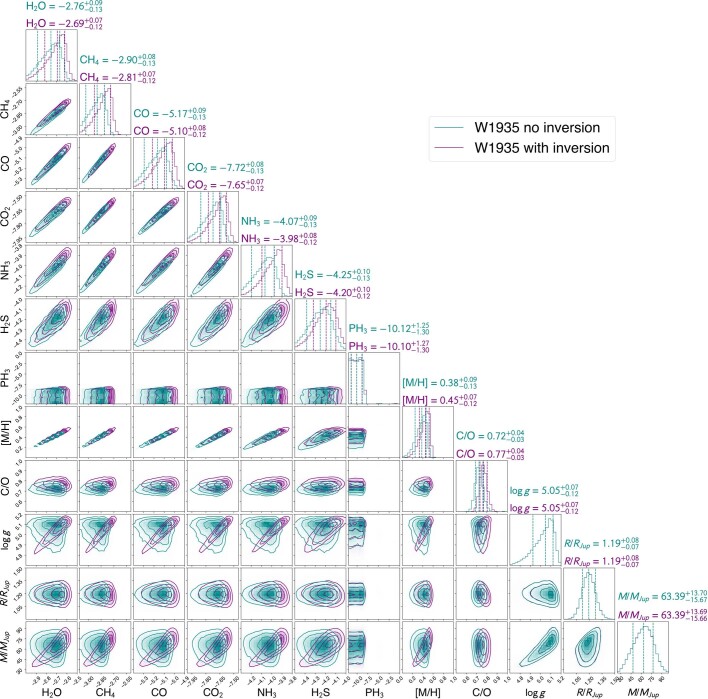
The retrieved composition of W1935, along with gravity, and derived quantities for mass, radius, metallicity and C/O. Posteriors for models with and without a temperature inversion are shown in purple and dark cyan respectively.

The posteriors for the non-temperature-related parameters for W1935 are also very similar between the two models, as shown in Extended Data Fig. 5. Although there are no significant differences between the compositions, there is a marginal trend across all the absorbing gases towards higher abundances in the baseline model (which allows for a temperature inversion).

Figures 2 and 3 demonstrate clearly that the model that allows a temperature inversion is the only one that is able to fit the CH_4_ emission feature at 3.326 μm, which is absent in the spectrum of W2220.

Our retrieval results for both objects show some differences from those inferred from our SED-based luminosity and evolutionary model predictions (Extended Data Figs. 5 and 2 and Extended Data Table 1). In both cases, the masses implied by our retrievals are slightly higher than the 1*σ* upper limit suggested by evolutionary models. The difference for W1935 is 1.3*σ*, and for W2220, it is 1.1*σ*. It is not unusual for retrieval analyses to disagree with evolutionary models’ predictions of log *g*, radius and mass, particularly in the absence of any strong prior evidence to provide empirical constraints^15,16,50,70^. In addition, these retrievals cover a relatively narrow wavelength range that lacks recognized gravity-sensitive spectral features. Establishing the presence and nature of any possible biases to higher gravity and mass estimates is beyond the scope of this work and does not impact our central results or conclusions.

**Extended Data Fig. 6:**
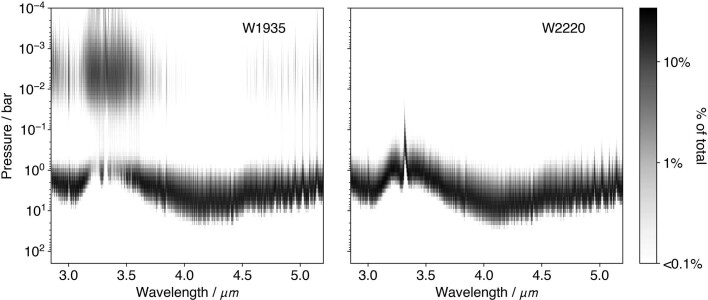
The contribution functions for W1935 and W2220 maximum likelihood retrieval models. The CH_4_ emission seen in W1935 originates from pressures shallower than 0.01 bar.

Extended Data Fig. 6 compares the contribution functions for maximum likelihood retrieval models for W1935 and W2220. The contribution function in an atmospheric layer lying between pressures *P*_1_ and *P*_2_ is defined as:

4 $$C(\lambda ,P)=\frac{B(\lambda ,T(P)){\int }_{{P}_{1}}^{{P}_{2}}\,{\rm{d}}\tau }{\exp {\int }_{0}^{{P}_{2}}\,{\rm{d}}\tau }.$$

*C*(*λ*, *P*) effectively maps the depth in the atmosphere from which the flux observed at a given wavelength originates. Extended Data Fig. 6 demonstrates that the CH_4_ emission seen in W1935 originates from pressures shallower than 0.01 bar. Our model finds gas some 300 K hotter than is retrieved when no inversion is permitted.

For simplicity, we used only cloud-free retrieval models. It has been well documented that the omission of clouds in a retrieval of an atmosphere that contains clouds can bias the thermal profile to a more isothermal gradient, as the retrieval mimics the effect of clouds in screening hotter deeper layers in the opacity windows between molecular absorption features^15,16,70^. Hence, the kinks seen in the retrieved thermal profiles may be suggestive that clouds should be considered for future retrieval studies in this temperature regime.

## Online content

Any methods, additional references, Nature Portfolio reporting summaries, source data, extended data, supplementary information, acknowledgements, peer review information; details of author contributions and competing interests; and statements of data and code availability are available at 10.1038/s41586-024-07190-w.

## Figures and Tables

**Extended Data Table 1:**
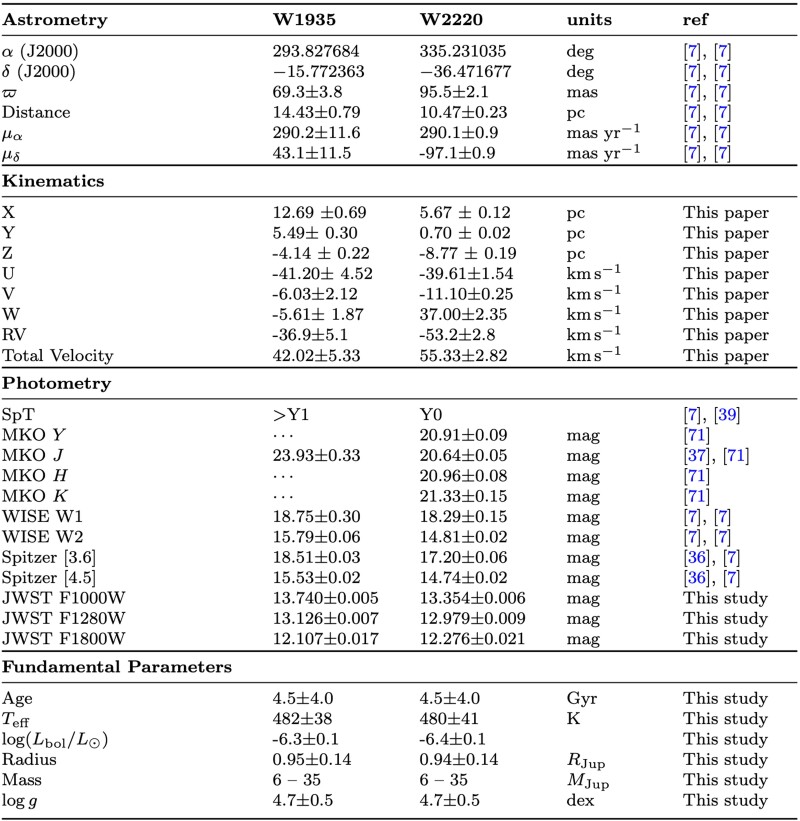
Parameters of interest for W1935 and W2220

Data from refs. ^7,36,37,39,71^.

**Extended Data Table 2:**
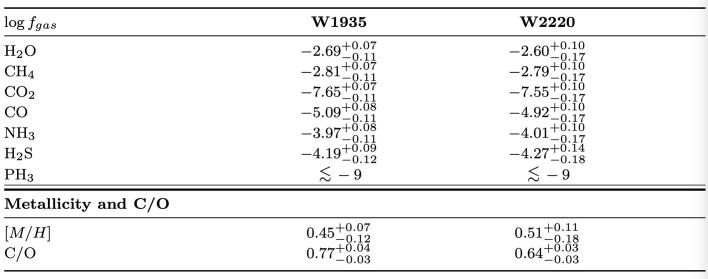
Retrieved Gas Abundances and Fundamental Parameters

**Extended Data Table 3:**
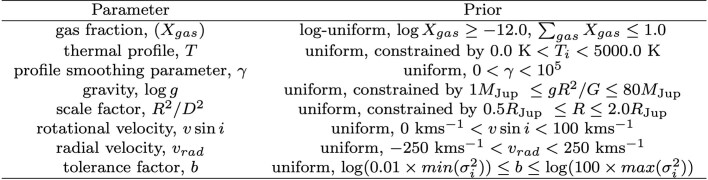
Parameters and priors adopted for the retrieval analysis

**Extended Data Table 4:**
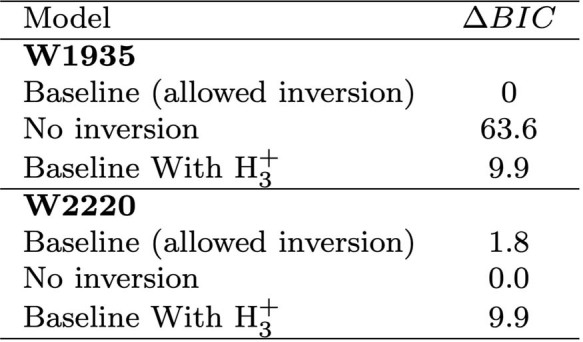
Δ*BIC* values for the models tested for each target

## Data Availability

The JWST data in this paper are part of GO programme 2124 (principal investigator: J.K.F.) and are publicly available from MAST (archive.stsci.edu/) under that programme ID. The HST WFC3 spectrum of W2220 is available from ui.adsabs.harvard.edu/abs/2015ApJ...804...92S/abstract.

## Code availability

The data reduction pipeline jwst can be found at jwst-pipeline.readthedocs.io/en/latest/. The Brewster code is open source and available at the following GitHub repository: github.com/substellar/brewster. Similarly, the SEDkit code is open source and available at github.com/hover2pi/sedkit. The set-up that yields the results presented herein is discussed in the Methods.
